# Morphology of Dbx1 respiratory neurons in the preBötzinger complex and reticular formation of neonatal mice

**DOI:** 10.1038/sdata.2017.97

**Published:** 2017-08-01

**Authors:** Victoria T. Akins, Krishanthi Weragalaarachchi, Maria Cristina D. Picardo, Ann L. Revill, Christopher A. Del Negro

**Affiliations:** 1 Department of Applied Science, The College of William and Mary, Williamsburg, Virginia 23185, USA; 2 Department of Physiology, Neuroscience and Mental Health Institute, Women and Children’s Health Research Institute, Faculty of Medicine and Dentistry, University of Alberta, Edmonton, Alberta T6G 2E1, Canada

**Keywords:** 3-D reconstruction, Neuroscience, Confocal microscopy, Nervous system

## Abstract

The relationship between neuron morphology and function is a perennial issue in neuroscience. Information about synaptic integration, network connectivity, and the specific roles of neuronal subpopulations can be obtained through morphological analysis of key neurons within a microcircuit. Here we present morphologies of two classes of brainstem respiratory neurons. First, interneurons derived from Dbx1-expressing precursors (Dbx1 neurons) in the preBötzinger complex (preBötC) of the ventral medulla that generate the rhythm for inspiratory breathing movements. Second, Dbx1 neurons of the intermediate reticular formation that influence the motor pattern of pharyngeal and lingual movements during the inspiratory phase of the breathing cycle. We describe the image acquisition and subsequent digitization of morphologies of respiratory Dbx1 neurons from the preBötC and the intermediate reticular formation that were first recorded *in vitro*. These data can be analyzed comparatively to examine how morphology influences the roles of Dbx1 preBötC and Dbx1 reticular interneurons in respiration and can also be utilized to create morphologically accurate compartmental models for simulation and modeling of respiratory circuits.

## Background & Summary

Neuronal morphology, particularly the structure of the dendritic tree, influences how a neuron integrates synaptic inputs and generates physiological output patterns. Axon projections provide information about connectivity patterns in microcircuits. This study documents the morphology of brainstem interneurons that generate and control breathing.

Breathing is a rhythmic motor behavior that ventilates the lungs to support respiration and homeostasis in air-breathing vertebrates. For humans and all mammals, rodents serve as an advantageous model systesm to study the neural origins of breathing. Key interneuron populations that generate inspiratory, expiratory, and (very recently) post-inspiratory related rhythms have been characterized in terms of physiology, genetic background, and transmitter phenotype^1,2,3,4^. Premotor neurons that influence airway resistance have been similarly characterized^5,6,7,8,9,10,11^. However, only a limited number of morphologies of constituent neurons in these populations have been documented and analyzed^5,12,13^. This data descriptor aims to ameliorate that problem by providing annotated, high-quality digital reconstructions of the morphologies of rhythm-generating interneurons and motor pattern-related premotor neurons from neonatal mice.

The respiratory cycle is dominated by the rhythm underlying inspiration, which is generated within the preBötzinger complex (preBötC) of the ventral medulla^1,2,3,4,14,15^. Rhythmogenic preBötC neurons are derived from precursor cells that express the homeobox transcription factor *Dbx1* (refs 13, 16,17,18), hereafter referred to as Dbx1 neurons. The intermediate reticular formation, immediately adjacent (dorsal) to the preBötC, is a diverse region containing respiratory Dbx1 premotor interneurons that control inspiratory related muscles of the tongue and pharynx^9,19,20,21^.

In this study we used intersectional mouse genetics to induce fluorescent protein expression in Dbx1 neurons of neonatal mice. Neuronal morphologies were acquired following patch-clamp recordings in transverse brainstem slices that retain the preBötC, the intermediate reticular formation, as well as the hypoglossal (XII) motor nucleus. These slices expose the preBötC and reticular formation at the rostral surface and spontaneously generate inspiratory rhythm and XII motor output, thus providing an experimentally advantageous breathing model *in vitro*^22,23^.

We obtained three-dimensional morphologies of respiratory Dbx1 preBötC and Dbx1 intermediate reticular formation neurons by filling neurons with biocytin during whole-cell patch-clamp recordings^24,25,26,27^. Compared to other reconstruction methods such as fluorescence microscopy of dye-filled neurons, biocytin reconstructions can be more time consuming but provide better visualization of thinner neuronal processes and axons^28^. Once labeled, we visualized the recorded neurons via confocal imaging and manually reconstructed their morphologies in a convenient digital format suitable for storage, display, and analysis.

Over the past four years, our laboratory contributed 47 digital neuronal morphologies to the public open access database NeuroMorpho.org. Of those 47 digital reconstructions, 23 correspond to Dbx1 preBötC neurons^12,13^ (six have not been previously published; this report describes them for the first time). Twelve of the 47 correspond to preBötC neurons not derived from *Dbx1*-expressing precursors^12^ (i.e., non-Dbx1 preBötC neurons), and 12 correspond to Dbx1 reticular formation neurons^5^.

Digital morphologies can be analyzed by software packages such as L-measure^29^, which computes more than 40 different morphometric properties of dendritic trees and axons. Sholl analysis, which provides branching and dendritic density information in regular distance intervals from the soma^30,31^, can be performed with software such as NeuronStudio^32^. Digital morphologies can also be readily ported to simulation packages such as NEURON^33^ and GENESIS^34^ to form compartmental mathematical models that are high-fidelity representations of real neurons. We intend that these morphological data be meta-analyzed and incorporated into models of inspiratory rhythm- and pattern-generating circuits of the lower brainstem to better understand the neural mechanisms of breathing.

## Methods

### Mice

All of the animal protocols were approved by the Institutional Animal Care and Use Committee at The College of William and Mary, which follows the guidelines provided by the US National Institutes of Health Office of Laboratory Animal Welfare^35^.

**Figure 1:**
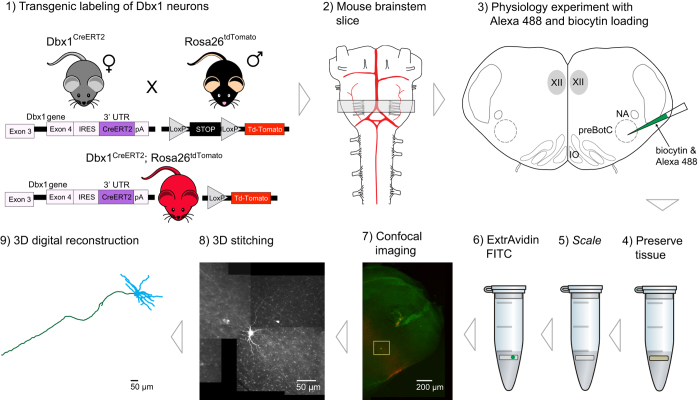
Workflow diagram for digital neuronal reconstructions. Expression of the red fluorescent protein tdTomato is induced in Dbx1-derived neurons (and glia) in mice using intersectional mouse genetic technologies (1). A transverse slice of the brainstem (indicated by the gray box) containing the preBötzinger complex is taken from a neonatal transgenic mouse (2). The slice is used for physiology recordings, during which respiratory modulated neurons are filled with biocytin (3). The slice is then preserved in 4% paraformaldehyde (4) and made transparent via incubation in Scale solution (5). The slice is treated with ExtrAvidin FITC (6) which binds to the biocytin allowing for visualization of the neuron through confocal microscopy (7). Confocal images in the x-, y-, and z- dimensions are taken of the entire neuron morphology and stitched together using FIJI (8). Using the 3D confocal images, neurons are digitally reconstructed using Neuromantic (9). XII, hypoglossal nucleus; NA, nucleus ambiguus, preBötC, preBötzinger complex; IO, inferior olive.

Figure 1 recaps the workflow, which is detailed below. We crossed female mice that express Cre recombinase fused to a tamoxifen-sensitive estrogen receptor (*CreER*^*T2*^) under the control of the *Dbx1* promoter, i.e., *Dbx1*^*CreERT2*^ (stock no. 028131, Jackson labs, Bar Harbor, ME)^36^ with floxed male reporter mice that express red fluorescent protein variant tdTomato in a Cre-dependent manner (*Rosa26*^*tdTomato*^, stock no. 007905, Jackson labs)^37^. Offspring with both alleles (*Dbx1*^*CreERT2*^*; Rosa26*^*tdTomato*^ mice), whose pregnant dams received tamoxifen during embryonic development, express the fluorescent reporter in Dbx1-derived cells^12,22,36^ (Fig. 1, step 1). *Dbx1*^*CreERT2*^ mice were maintained on a CD-1 background strain. *Rosa26*^*tdTomato*^ reporter mice were maintained using a C57BL/6J background strain.

*Dbx1*^*CreERT2*^ mice were also mated with floxed reporter mice that express a channelrhodopsin-2/tdTomato fusion protein (*Rosa26*^*ChR2-tdTomato*^, stock no. 12567, Jackson labs)^38^. The *Dbx1*^*CreERT2*^; *Rosa26*^*ChR2-tdTomato*^ mice were employed in separate electrophysiological experiments; here we recovered the morphology of the recorded neurons in the same way as *Dbx1*^*CreERT2*^*; Rosa26*^*tdTomato*^, which was possible because both expressed native tdTomato in Dbx1-derived neurons. Channelrhodopsin, while important for physiological tests, has no impact on morphological studies. Figure 1 only indicates *Dbx1*^*CreERT2*^*; Rosa26*^*tdTomato*^ mice for simplicity.

Animal genotypes were verified using real-time PCR using primers for *Cre* and tandem dimer red fluorescent protein (Transnetyx, Cordova, TN). Timed matings were monitored such that embryonic day 0.5 (E0.5) was defined as 12 h after the start of cohabitation. Cre recombination was then induced by administering tamoxifen (T5648; Sigma Aldrich, St Louis, MO) at E10.5 when *Dbx1* is at or near peak expression in the hindbrain^16,17,36,39^. Tamoxifen was administered by oral gavage to pregnant dams at a concentration of 0.9 mg/40 g body mass.

### Transverse slice preparations

Neonatal *Dbx1*^*CreERT2*^; *Rosa26*^*tdTomato*^ and *Dbx1*^*CreERT2*^*; Rosa26*^*ChR2-tdTomato*^ mice were anesthetized then euthanized via decapitation at postnatal days 0–5 (P0–5), consistent with protocols outlined by the American Veterinary Medical Association Guidelines for euthanasia of animals^40^. Transections were made at the bregma and the thorax. The neuraxis, from the pons to the lower thoracic spinal cord, was then removed within two minutes and further dissected in artificial cerebrospinal fluid (ACSF) containing (mM): 124 NaCl, 3 KCl, 1.5 CaCl_2_, 1 MgSO_4_, 25 NaHCO_3_, 0.5 NaH_2_PO_4_ and 30 dextrose, equilibrated with 95% O_2_ and 5% CO_2_ (pH 7.4) (Fig. 1, step 2). The neuraxis was then glued to an agar block with the ventral surface facing out and placed in the vise of a vibratome. We cut 550-μm-thick transverse brainstem slices that exposed the preBötC at the rostral face and retained the rostral XII nerve rootlets^22^ (Fig. 1, step 3). Slices were perfused with ACSF at 28 °C in a recording chamber on a fixed-stage upright microscope equipped with differential interference contrast optics and epifluorescence, which enables visual identification and selective recording of target neurons. The K^+^ concentration in the ACSF was elevated to 9 mM to maintain long-term stability of the preBötC rhythm^22,23,41^. Rhythmic inspiratory-related motor output was recorded from the XII nerve rootlets using suction electrodes and a differential amplifier. Whole-cell patch-clamp recordings were acquired using capillary glass micro-pipettes and a current-clamp amplifier. Patch pipettes were positioned under visual control after fluorescent identification of Dbx1 neurons. The patch solution contained (mM): 140 potassium gluconate, 10 Hepes, 5 NaCl, 1 MgCl_2_, 0.1 EGTA, 2 Mg-ATP, 0.3 Na3-GTP and 2 mgml^−1^ biocytin (B4261; Sigma Aldrich). All of the neurons in this data set were rhythmically active in sync with inspiratory XII motor output.

After the recordings, transverse slices containing biocytin-filled neurons were fixed in 4% paraformaldehyde in 0.1 M sodium phosphate buffer for at least 16 h at 4 °C (Fig. 1, step 4). Then, the slices were treated with *Scale* solution containing 4 M urea, 10% (mass/volume) glycerol and 0.1% (m/v) Triton X-100, for 10 days to clear the tissue and remove opaque background staining^42^ (Fig. 1, step 5). Slices were washed three times for 15 min each in phosphate buffer solution (PBS)+1% Triton X-100 (PBST) and then blocked in PBST with 10% heat-inactivated fetal bovine sera (F4135; Sigma Aldrich) for 45 min. The biocytin was revealed by incubating the slices with fluorescein isothiocyanate-conjugated ExtrAvidin (E2761; Sigma Aldrich) overnight at 4 °C with three-dimensional rotation on a nutator (Fig. 1, step 6). Next, the slices were rinsed with PBS five times for 15 min each and cover-slipped in Vectashield (H-1500; Vector Laboratories, Burlingame, CA).

### Confocal microscopy and digital neuronal reconstruction

We visualized recorded neurons using a spinning-disk confocal microscope (Olympus BX51, Center Valley, PA) and a laser scanning confocal microscope (Zeiss LSM 510, Thornwood, NY) Three-dimensional (3D) confocal images of the individual neurons were obtained using a 20x objective (Olympus numerical aperture 0.5, Zeiss LSM numerical aperture 1.0) at increments of 1 μm in the z-axis (Fig. 1, step 7). The series of confocal images (i.e., z-stacks) were aligned in three-dimensions, merged or ‘stitched together’ at contiguous borders using ImageJ software^43^ and the Stitching plugin^44^ (Fig. 1, step 8). This stitching process was iterated until the entire morphology of the neuron was contained within a single three-dimensional image file. Finally we digitized neuronal morphologies using the Neuromantic reconstruction tool, which is also free and in the public domain^45^. The digital reconstructions were scaled to the appropriate size based on the micron-to-pixel ratio for each microscope (Fig. 1, step 9). Images acquired from the LSM microscope were scaled with a 0.41 micron-to-pixel ratio and images from the Olympus microscope were scaled using a 0.322 micron-to-pixel ratio. This data descriptor pertains to 47 digital morphologies of inspiratory modulated Dbx1 preBötC neurons, six of which are previously unpublished (Data Citations 1–6) and 41 which are associated with previous publications (Data Citations 7–47). The morphologies are all publicly available via NeuroMorpho.org.

## Data Records

Digital reconstructions of Dbx1 neurons are located in the Del Negro archive of the NeuroMorpho database (Data Citations 1–47). Digital reconstruction files are in SWC format, which is a commonly used format for neuron morphologies^24^. The reconstruction files contain an x-coordinate, y-coordinate, and z-coordinate of each neuronal segment. The type of neuronal process, such as cell body, axon, or dendrite is also specified by type 1, 2, and 4, respectively. (Type 3 represents basal dendrites, but there is no such distinction in brainstem interneurons, so type 3 is omitted as a classifier in our dataset. Our dendrites were all designated type 4.) The radius in microns is given for each neuronal segment as well as the ‘parent’ segment or the index number of the previous segment. Table 1 provides an example of an SWC file output for a neuron reconstruction. Physiological properties of Dbx1 preBötC and Dbx1 reticular neurons have been described^5,12,13^. Table 2 lists the reconstructions available in the Del Negro archive of NeuroMorpho.org.

## Technical Validation

In newborn *Dbx1*^*CreERT2*^; *Rosa26*^*tdTomato*^ and *Dbx1*^*CreERT2*^*; Rosa26*^*ChR2-tdTomato*^ mice, Dbx1 neurons form an inverted U-shape in the transverse (coronal) plane, which is visible in brainstem slices at the level of the preBötC. The inverted U-shape originates at the lateral border of the hypoglossal motor nucleus, located within the dorso-medial portion of the slice, and continues ventrolaterally until the ventral border of the tissue slice^22^. The dorsal border of the preBötC is identifiable because it is immediately ventral to the semi-compact division of the nucleus ambiguus, which does not express Dbx1^22^. Visual identification of the principal loop of the inferior olive and the flattening of the V-shape of the fourth ventricle are other indicators that the rostral surface of the transverse slice is at the level of the preBötC^22^.

Slices remained in the recording chamber for at least 15 min after biocytin dialysis to maximize biocytin diffusion throughout the cytoplasm^12,24^. A clearing agent was used to facilitate visualization of the morphology; however clearing reagents can cause tissue shrinkage or expansion which could distort morphological features^42^. The *Scale* solution used to clear the tissue in these experiments minimizes or completely precludes tissue expansion (compared to other methods)^42^.

The quality of digital reconstructions depends on histology methods, image acquisition, as well as the digital reconstruction algorithms. To minimize disparities, we consistently used the same method of histological labeling. The software Neuromantic^45^, used for digitizing our image stacks, offers up to 16,000% magnification. This zoom feature enables the user to adhere to the most minute details captured in the image, which results in the most accurate reconstruction possible.

Two of the six new neurons and nine of the previously published neurons had no discernible axon, which might have indicated insufficient biocytin filling or that the axon was severed during tissue preparation. We recommend that the end user of the data draw no firm conclusions regarding connectivity from the lack of an axon in reconstructed digital morphology.

For those neurons whose axons were discernible, we distinguished the axons from the dendrites according to these criteria: 1) axons generally have a constant diameter whereas dendrites taper distal to the soma; 2) axons exhibit fewer branches and never show spine-like protrusions; 3) truncated axons near the slice surface exhibit a bleb or fluorescent circle from the cut end^46,47^.

Digital reconstructions were uploaded to NeuroMorpho.org, where they undergo a standardization process. The soma (type 1) should be the initial parent segment for all subsequent segments, whether dendritic or axonal. Neuronal processes should only connect to either the soma or to segments of the same type; for example, dendrite segments connect to dendrite segments and axon segments connect to axon segments. All processes should have a designated type and should not be undefined. A process can branch into no more than two processes at any given point.

Some irregularities can be fixed automatically during the standardization process^48^. If a neuronal segment is designated as a different type than its parent and daughter segments (e.g., a type 3 surrounded by type 2 s) the erroneous segment type is automatically changed to match the type of its parent and daughter segments. If the soma is not the initial segment in the file, the soma segment is automatically changed to the first segment in the file. If a segment has a radius of zero microns, then the radius is automatically changed to match the radius of its parent. Other digitization issues must be corrected by the submitting investigator^48^. For example, if a segment has not been designated with a process type, the correct type must be manually entered, rather than automatically assigned, which ensures that the proper type has been documented. Segments with a radius of zero (i.e., less than 0.05 μm), or larger than four standard deviations above the average radius of the cell are flagged as physiologically unrealistic during standardization and must be resolved by the submitting investigator. After the standardization process, digital reconstruction files and images are then reviewed and approved by the submitting investigator before being added to the public database^48^.

## Additional Information

**How to cite this article:** Akins, V. T. *et al.* Morphology of Dbx1 respiratory neurons in the preBötzinger complex and reticular formation of neonatal mice. *Sci. Data* 4:170097 doi: 10.1038/sdata.2017.97 (2017).

**Publisher’s note:** Springer Nature remains neutral with regard to jurisdictional claims in published maps and institutional affiliations.

## Supplementary Material



## Figures and Tables

**Table 1:** Sample SWC file data for digital neuron reconstruction.

**Index**	**Type**	**x**	**y**	**z**	**Radius**	**Parent**
1	1	967.7	1539.0	9	13.81	−1
2	4	970.0	1533.0	10	7.35	1
3	4	971.3	1530.5	10	4.59	2
4	4	972.0	1528.5	11	3.58	3
5	4	972.1	1525.2	11	3.27	4
6	4	971.9	1522.6	11	2.57	5
7	4	971.6	1520.1	11	2.17	6
8	4	971.1	1517.3	10	1.77	7
9	4	971.0	1514.7	9	1.68	8
10	4	970.4	1513.3	9	1.61	9
11	4	969.2	1510.2	9	1.50	10
12	4	968.5	1509.6	9	1.50	11
13	4	967.5	1509.4	9	1.50	12
14	4	966.9	1507.9	8.5	1.50	13
15	4	966.5	1506.2	8	1.50	14
16	4	966.3	1504.9	8	1.50	15
17	4	965.9	1503.5	8	1.49	16
18	4	965.3	1502.5	8	1.50	17
19	4	965.1	1501.2	8	1.50	18
20	4	965.9	1500.5	8	1.46	19
21	4	966.0	1499.2	8	1.43	20
22	4	966.1	1496.0	8	1.38	21
23	4	965.2	1494.5	7	1.31	22
24	4	964.7	1493.3	7	1.32	23
25	4	964.0	1492.1	7	1.35	24
26	4	963.6	1490.7	7	1.33	25
27	4	963.0	1489.2	7	1.40	26
28	4	962.6	1488.1	7	1.39	27
29	4	962.5	1486.6	7	1.34	28
30	4	962.5	1485.3	7	1.32	29
31	4	962.1	1483.9	7	1.41	30
32	4	961.6	1481.6	7	1.32	31
33	4	961.8	1480.3	7	1.34	32
34	4	961.9	1479.6	7	1.33	33
35	4	962.0	1479.0	7	1.33	34
36	4	962.4	1478.0	7	1.30	35
37	4	962.4	1476.7	7	1.27	36
http://www.reading.ac.uk/neuromantic/body_index.php.						

**Table 2:** Digital reconstructions in NeuroMorpho.Org Del Negro Lab Archive.

**Neuron Name**	**Cell type**	**Location**	**NeuroMorpho ID**
120621_Dbx1PreBotC	Dbx1	preBötC	NMO_45917
120623_Dbx1PreBotC	Dbx1	preBötC	NMO_45918
130819_Dbx1PreBotC	Dbx1	preBötC	NMO_45921
130910_Dbx1PreBotC	Dbx1	preBötC	NMO_45922
140110_Dbx1PreBotC	Dbx1	preBötC	NMO_45923
140117_Dbx1PreBotC	Dbx1	preBötC	NMO_45924
130212_Dbx1PreBötC1	Dbx1	preBötC	NMO_45919
130212_Dbx1PreBötC2	Dbx1	preBötC	NMO_45920
140127_Dbx1PreBötC	Dbx1	preBötC	NMO_45925
111220-Dbx1-2	Dbx1	preBötC	NMO_09581
111220-Dbx1-1	Dbx1	preBötC	NMO_09582
111219-Dbx1-S2	Dbx1	preBötC	NMO_09583
111219-Dbx1-S1	Dbx1	preBötC	NMO_09584
111214-Dbx1	Dbx1	preBötC	NMO_09585
111212-Dbx1	Dbx1	preBötC	NMO_09586
111209-Dbx1	Dbx1	preBötC	NMO_09587
111208-Dbx1	Dbx1	preBötC	NMO_09588
111109-Dbx1-2	Dbx1	preBötC	NMO_09589
111109-Dbx1-1	Dbx1	preBötC	NMO_09590
111108-Dbx1	Dbx1	preBötC	NMO_09591
111102-Dbx1-2	Dbx1	preBötC	NMO_09592
111102-Dbx1-1	Dbx1	preBötC	NMO_09593
111101-Dbx1	Dbx1	preBötC	NMO_09594
111222-ctrl-2	Dbx1	preBötC	NMO_09595
111222-ctrl-1	Dbx1	preBötC	NMO_09596
111220-ctrl	non-Dbx1	preBötC	NMO_09597
111219-ctrl-2	non-Dbx1	preBötC	NMO_09598
111219-ctrl-1	non-Dbx1	preBötC	NMO_09599
111214-ctrl-2	non-Dbx1	preBötC	NMO_09600
111214-ctrl-1	non-Dbx1	preBötC	NMO_09601
111209-ctrl	non-Dbx1	preBötC	NMO_09602
111208-ctrl	non-Dbx1	preBötC	NMO_09603
111103-ctrl	non-Dbx1	preBötC	NMO_09604
111101-ctrl	non-Dbx1	preBötC	NMO_09605
111031-ctrl	non-Dbx1	preBötC	NMO_09606
130624_Dbx1RF	Dbx1	Reticular formation	NMO_45926
130625_Dbx1RF	Dbx1	Reticular formation	NMO_45927
140109_Dbx1RF	Dbx1	Reticular formation	NMO_45928
140114_Dbx1RF	Dbx1	Reticular formation	NMO_45929
140120_Dbx1RF	Dbx1	Reticular formation	NMO_45930
140124_Dbx1RF	Dbx1	Reticular formation	NMO_45931
140207_Dbx1RF	Dbx1	Reticular formation	NMO_45932
140208_Dbx1RF	Dbx1	Reticular formation	NMO_45933
140220_Dbx1RF	Dbx1	Reticular formation	NMO_45934
140221_Dbx1RF	Dbx1	Reticular formation	NMO_45935
140301_Dbx1RF	Dbx1	Reticular formation	NMO_45936
140306_Dbx1RF	Dbx1	Reticular formation	NMO_45937

## References

[d1] NeuroMorpho.org Del Negro C. A. 2017 NMO_45917

[d2] NeuroMorpho.org Del Negro C. A. 2017 NMO_45918

[d3] NeuroMorpho.org Del Negro C. A. 2017 NMO_45921

[d4] NeuroMorpho.org Del Negro C. A. 2017 NMO_45922

[d5] NeuroMorpho.org Del Negro C. A. 2017 NMO_45923

[d6] NeuroMorpho.org Del Negro C. A. 2017 NMO_45924

[d7] NeuroMorpho.org Del Negro C. A. 2013 NMO_09581

[d8] NeuroMorpho.org Del Negro C. A. 2013 NMO_09582

[d9] NeuroMorpho.org Del Negro C. A. 2013 NMO_09583

[d10] NeuroMorpho.org Del Negro C. A. 2013 NMO_09584

[d11] NeuroMorpho.org Del Negro C. A. 2013 NMO_09585

[d12] NeuroMorpho.org Del Negro C. A. 2013 NMO_09586

[d13] NeuroMorpho.org Del Negro C. A. 2013 NMO_09587

[d14] NeuroMorpho.org Del Negro C. A. 2013 NMO_09588

[d15] NeuroMorpho.org Del Negro C. A. 2013 NMO_09589

[d16] NeuroMorpho.org Del Negro C. A. 2013 NMO_09590

[d17] NeuroMorpho.org Del Negro C. A. 2013 NMO_09591

[d18] NeuroMorpho.org Del Negro C. A. 2013 NMO_09592

[d19] NeuroMorpho.org Del Negro C. A. 2013 NMO_09592

[d20] NeuroMorpho.org Del Negro C. A. 2013 NMO_09594

[d21] NeuroMorpho.org Del Negro C. A. 2013 NMO_09595

[d22] NeuroMorpho.org Del Negro C. A. 2013 NMO_09596

[d23] NeuroMorpho.org Del Negro C. A. 2013 NMO_09597

[d24] NeuroMorpho.org Del Negro C. A. 2013 NMO_09598

[d25] NeuroMorpho.org Del Negro C. A. 2013 NMO_09599

[d26] NeuroMorpho.org Del Negro C. A. 2013 NMO_09600

[d27] NeuroMorpho.org Del Negro C. A. 2013 NMO_09601

[d28] NeuroMorpho.org Del Negro C. A. 2013 NMO_09602

[d29] NeuroMorpho.org Del Negro C. A. 2013 NMO_09603

[d30] NeuroMorpho.org Del Negro C. A. 2013 NMO_09604

[d31] NeuroMorpho.org Del Negro C. A. 2013 NMO_09605

[d32] NeuroMorpho.org Del Negro C. A. 2013 NMO_09606

[d33] NeuroMorpho.org Del Negro C. A. 2016 NMO_45919

[d34] NeuroMorpho.org Del Negro C. A. 2016 NMO_45920

[d35] NeuroMorpho.org Del Negro C. A. 2016 NMO_45925

[d36] NeuroMorpho.org Del Negro C. A. 2016 NMO_45926

[d37] NeuroMorpho.org Del Negro C. A. 2016 NMO_45927

[d38] NeuroMorpho.org Del Negro C. A. 2016 NMO_45928

[d39] NeuroMorpho.org Del Negro C. A. 2016 NMO_45929

[d40] NeuroMorpho.org Del Negro C. A. 2016 NMO_45930

[d41] NeuroMorpho.org Del Negro C. A. 2016 NMO_45931

[d42] NeuroMorpho.org Del Negro C. A. 2016 NMO_45932

[d43] NeuroMorpho.org Del Negro C. A. 2016 NMO_45933

[d44] NeuroMorpho.org Del Negro C. A. 2016 NMO_45934

[d45] NeuroMorpho.org Del Negro C. A. 2016 NMO_45935

[d46] NeuroMorpho.org Del Negro C. A. 2016 NMO_45936

[d47] NeuroMorpho.org Del Negro C. A. 2016 NMO_45937

